# Supplementary material for: Leucine-Rich Glioma-Inactivated 1 (LGI1) Protein Stimulates Proliferation and IL-10 Production in Peripheral Blood Mononuclear Cells of Patients with LGI1 Antibody-Mediated Autoimmune Encephalitis In Vitro
Source: Int J Mol Sci. 2024 Feb 23;25(5):2581. doi: 10.3390/ijms25052581 (PMC10932227; doi:10.3390/ijms25052581)
Supplement: Supplementary file 1 [file ijms-25-02581-s001.zip › ijms-2769094-supplementary.pdf]

International Journal of *Molecular Sciences*

MDPI

**SUPPLEMENT FOR Goihl et al.**

**Table S1:** Sex and age data of 10 healthy control volunteers and 4 patients with LGI1 LE.

| | **Sex** | **Age** |
|---|---|---|
| healthy control 1 | Female | 36 |
| healthy control 2 | Female | 31 |
| healthy control 3 | Female | 63 |
| healthy control 4 | Male | 50 |
| healthy control 5 | Male | 54 |
| healthy control 6 | Female | 59 |
| healthy control 7 | Female | 59 |
| healthy control 8 | Male | 61 |
| healthy control 9 | Female | 54 |
| healthy control 10 | Male | 63 |
| patient 1 | Female | 60 |
| patient 2 | Female | 67 |
| patient 3 | Male | 61 |
| patient 4 | Female | 46 |

**Table S2.** Proliferation calculated as stimulation index of PBMC of 4 patients with LGI1 LE or of 10 healthy control volunteers in absence or presence of native or denatured recombinant LGI1 after 4 days of incubation.

| Proliferation (stimulation index) | control | supernatant | native LGI1 | denaturated LGI1 |
|---|---|---|---|---|
| healthy control 1 | 1 | 1,68 | 1,89 | 1,98 |
| healthy control 2 | 1 | 1,81 | 1,13 | 1,69 |
| healthy control 3 | 1 | 0,79 | 0,71 | 1,13 |
| healthy control 4 | 1 | 2,94 | 4,11 | 0,92 |
| healthy control 5 | 1 | 0,82 | 0,83 | 0,96 |
| healthy control 6 | 1 | 1,72 | 1,11 | 2,33 |
| healthy control 7 | 1 | 1,09 | 1,59 | 2,19 |
| healthy control 8 | 1 | 1,06 | 0,99 | 1,00 |
| healthy control 9 | 1 | 0,9 | 1,36 | 1,00 |
| healthy control 10 | 1 | 1,04 | 1,22 | 1,32 |
| patient 1 | 1 | 0,81 | 0,94 | 0,99 |
| patient 2 | 1 | 1,38 | 6,32 | 5,18 |
| patient 3 | 1 | 1,31 | 6,50 | 4,67 |
| patient 4 | 1 | 1,25 | 27,61 | 13,73 |

**Table S3.** Concentrations of IFN-γ in cell culture supernatants of PBMC of 4 patients with LGI1 LE or of 10 healthy control volunteers in absence or presence of native or denatured recombinant LGI1 after 4 days of incubation.

| IFN-γ (pg/ml) | control | supernatant | native LGI1 | denaturated LGI1 |
|---|---|---|---|---|
| healthy control 1 | 21.0 | 17.2 | 68.9 | 28.6 |
| healthy control 2 | 23.0 | 9.1 | 14.8 | 25.8 |
| healthy control 3 | 20.5 | 23.5 | 31.6 | 40.9 |
| healthy control 4 | 20.1 | 11.9 | 58.1 | 29.4 |
| healthy control 5 | 20.7 | 34.1 | 233.2 | 50.4 |
| healthy control 6 | 9.6 | 8.8 | 9.7 | 10.8 |
| healthy control 7 | 9.1 | 8.1 | 15.1 | 12.8 |
| healthy control 8 | 7.2 | 19.8 | 40.0 | 10.8 |
| healthy control 9 | 7.5 | 7.7 | 10.6 | 8.1 |
| healthy control 10 | 12.8 | 8.9 | 9.9 | 7.9 |
| patient 1 | 14.9 | 22.7 | 30.0 | 14.0 |
| patient 2 | 38.7 | 22.0 | 10.7 | 23.4 |
| patient 3 | 65.3 | 16.8 | 17.5 | 20.0 |
| patient 4 | 12.5 | 27.8 | 53.6 | 37.7 |

**Table S4.** Concentrations of IL-5 in cell culture supernatants of PBMC of 4 patients with LGI1 LE or of 10 healthy control volunteers in absence or presence of native or denatured recombinant LGI1 after 4 days of incubation.

| IL-5 (pg/ml) | control | supernatant | native LGI1 | denaturated LGI1 |
|---|---|---|---|---|
| healthy control 1 | 1.6 | 0.8 | 1.4 | 3.8 |
| healthy control 2 | 2.6 | 3.1 | 1.7 | 2.2 |
| healthy control 3 | 2.1 | 2.0 | 2.4 | 1.4 |
| healthy control 4 | 3.3 | 6.1 | 3.6 | 2.9 |
| healthy control 5 | 2.3 | 1.4 | 1.4 | 1.3 |
| healthy control 6 | 1.2 | 1.0 | 1.3 | 1.5 |
| healthy control 7 | 1.8 | 2.4 | 0.9 | 1.0 |
| healthy control 8 | 1.6 | 0.8 | 2.2 | 1.0 |
| healthy control 9 | 0.5 | 0.6 | 1.0 | 0.8 |
| healthy control 10 | 0.7 | 0.8 | 1.1 | 0.9 |
| patient 1 | 2.6 | 5.8 | 3.4 | 2.7 |
| patient 2 | 1.2 | 3.3 | 2.2 | 0.9 |
| patient 3 | 1.6 | 1.2 | 1.8 | 1.6 |
| patient 4 | 1.5 | 1.6 | 1.1 | 2.3 |

**Table S5.** Concentrations of IL-10 in cell culture supernatants of PBMC of 4 patients with LGI1 LE or of 10 healthy control volunteers in absence or presence of native or denatured recombinant LGI1 after 4 days of incubation.

| IL-10 (pg/ml) | control | supernatant | native LGI1 | denaturated LGI1 |
|---|---|---|---|---|
| healthy control 1 | 7.2 | n.d. | 538.5 | 460.2 |
| healthy control 2 | 2.5 | 6.6 | 11.4 | 6.8 |
| healthy control 3 | 2.2 | 14.1 | 25.7 | 21.2 |
| healthy control 4 | 2.9 | 33.4 | 427.0 | 85.9 |
| healthy control 5 | 129.1 | 136.7 | 206.0 | 219.4 |
| healthy control 6 | 129.5 | 121.4 | 127.8 | 124.7 |
| healthy control 7 | 126.5 | 131.3 | 244.5 | 162.7 |
| healthy control 8 | 125.9 | 127.5 | 147.6 | 132.5 |
| healthy control 9 | 126.2 | 125.3 | 127.5 | 124.4 |
| healthy control 10 | 124.5 | 125.6 | 124.8 | 125.0 |
| patient 1 | 13.6 | n.d. | 1149.2 | 871.0 |
| patient 2 | 4.7 | 37.6 | 2071.2 | 432.1 |
| patient 3 | 5.3 | 16.4 | 1594.3 | 205.3 |
| patient 4 | 8.8 | 22.5 | 1022.8 | 249.9 |

**Table S6.** Concentrations of IL-17 in cell culture supernatants of PBMC of 4 patients with LGI1 LE or of 10 healthy control volunteers in absence or presence of native or denatured recombinant LGI1 after 4 days of incubation.

| IL-17 (pg/ml) | control | supernatant | native LGI1 | denaturated LGI1 |
|---|---|---|---|---|
| healthy control 1 | 7.9 | 8.8 | 15.5 | 14.1 |
| healthy control 2 | 12.7 | 12.8 | 13.2 | 14.4 |
| healthy control 3 | 11.8 | 12.9 | 12.4 | 12.4 |
| healthy control 4 | 12.2 | 13.9 | 40.2 | 40.2 |
| healthy control 5 | 2.5 | 5.0 | 5.7 | 9.3 |
| healthy control 6 | 4.2 | 2.8 | 2.5 | 2.4 |
| healthy control 7 | 2.7 | 3.3 | 0.0 | 3.5 |
| healthy control 8 | 3.4 | 31.6 | 5.9 | 2.5 |
| healthy control 9 | 1.3 | 1.5 | 1.9 | 1.9 |
| healthy control 10 | 3.3 | 5.0 | 0.9 | 1.6 |
| patient 1 | 8.8 | 20.5 | 44.2 | 26.2 |
| patient 2 | 12.4 | 12.1 | 13.3 | 13.1 |
| patient 3 | 11.8 | 12.5 | 13.1 | 13.1 |
| patient 4 | 7.3 | 6.7 | 20.5 | 6.1 |

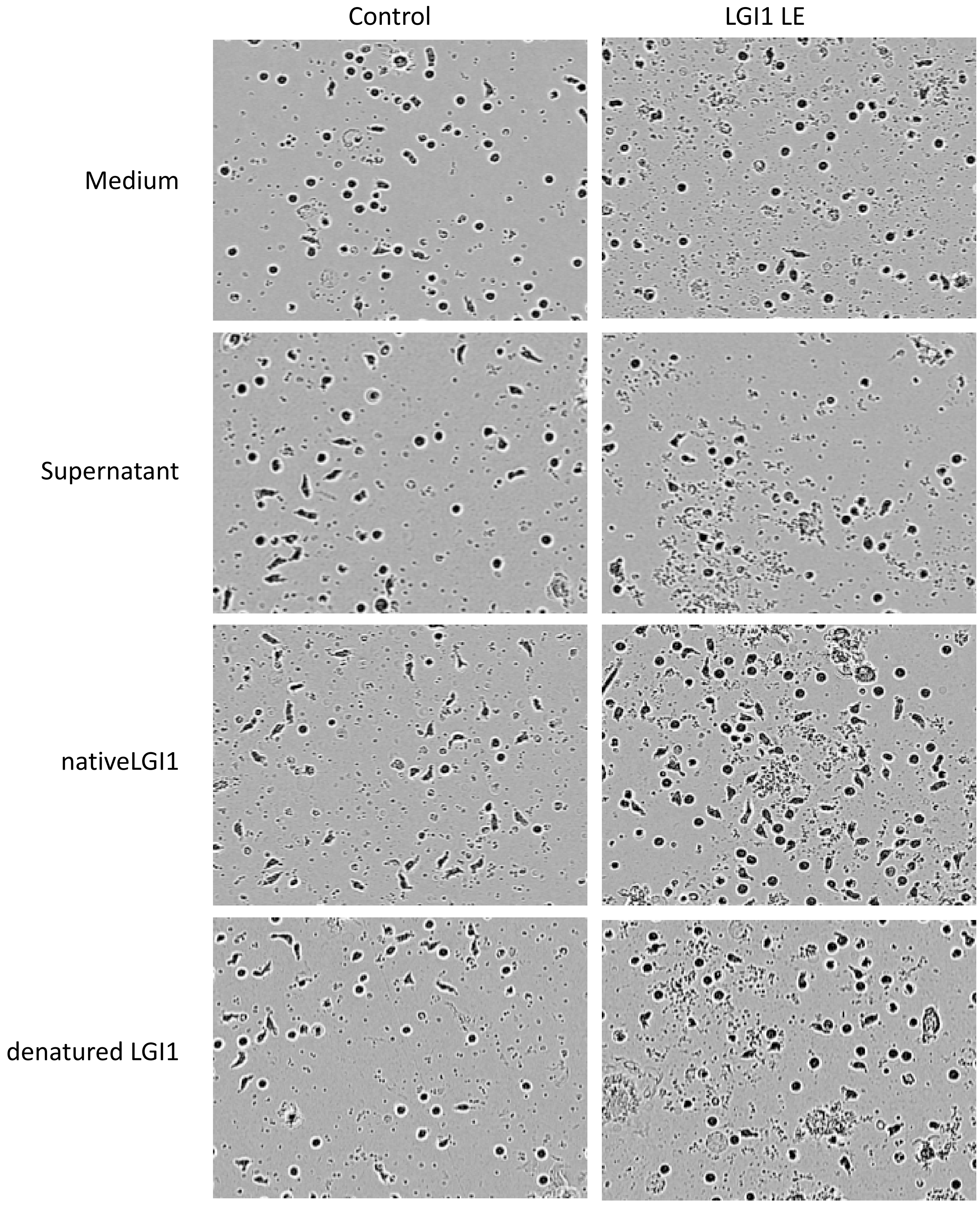


**Figure S1.** Representative pictures of cell cultures of PBMC from a healthy control and a patient with LGI1 ab-mediated LE incubated with medium, HEK 293 cell supernatant, native and denatured LGI1.

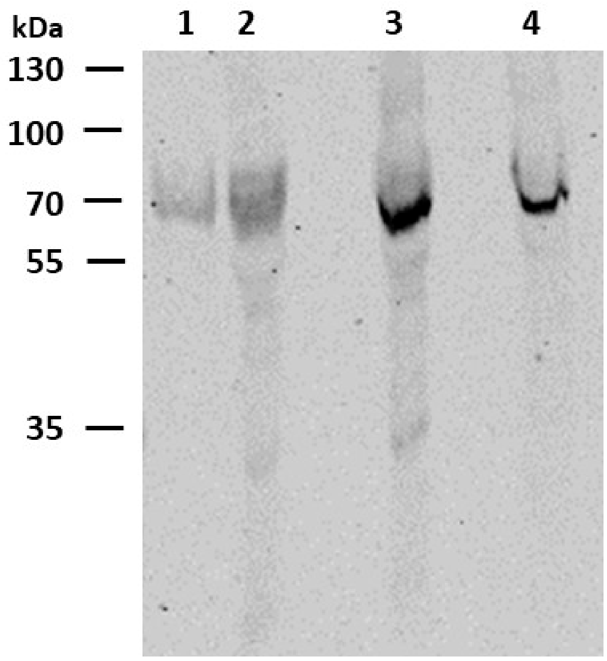


**Figure S2.** Verification of LGI1 expression and purification: Lane 1: ahead of every LGI1 purification protein expression and secretion was checked in supernatants by SDS-PAGE with Coomassie staining. Lane 2: after elution from His-Tag affinity chromatography increased concentration of LGI1 could be detected. Lane 3 and 4: polishing LGI1-containing solution with always 2 runs of size exclusion chromatography improved the purity of the desired protein.
